# FINANCIAL HARDSHIPS AND MEDICATION ADHERENCE DURING THE COVID-19 PANDEMIC

**DOI:** 10.1093/geroni/igac059.2983

**Published:** 2022-12-20

**Authors:** Megan Lytle, Dawn Carr, Patricia Homan

**Affiliations:** Florida State University, Tallahassee, Florida, United States; Florida State University, Tallahassee, Florida, United States; Florida State University, Tallahassee, Florida, United States

## Abstract

Medication nonadherence is associated with numerous negative health outcomes among older adults, including myocardial infarction, stroke, preventable hospitalizations, and increased risk of decline in self-reported health status. Maintaining continuous use and access to needed medications in later life has important implications for quality and length of life. A primary barrier shown to interfere with medication adherence in older adults is an inability to pay for medication. Relative to their younger counterparts, older adults have more financial protections that increase access to needed prescription medication through health insurance coverage. Despite these added protections, older adults are more likely to experience financial insecurity, with some evidence suggesting that COVID-19 accentuated existing vulnerabilities. Data are derived from the Health and Retirement Study (HRS), leveraging data drawn from the 2016, 2018, and 2020 study waves (n=3,185). Logistic models were used to evaluate the association between five COVID-19 related financial setbacks (i.e., inability to pay mortgage/rent, credit card bills, utility or insurance bills, medical bills, and inadequate money for food), and medication nonadherence among adults 60+. Results show that net of pre-COVID financial vulnerabilities and socioeconomic status, individuals who reported being unable to pay medical bills and those unable to pay rent/mortgage after the start of the pandemic reported higher odds (19% higher and 230% higher odds, respectively) of not taking/filling their prescription medication due to cost. Results suggest that greater financial protections for housing and medical bills among financially vulnerable older adults will increase medication adherence.

